# Norcantharidin reverses cisplatin resistance and inhibits the epithelial mesenchymal transition of human non-small lung cancer cells by regulating the YAP pathway

**DOI:** 10.3892/or.2020.7830

**Published:** 2020-11-02

**Authors:** Dan Jin, Yan Wu, Cuijie Shao, Yong Gao, Deqiang Wang, Jiwei Guo

Oncol Rep 40: 609-620, 2018; DOI: 10.3892/or.2018.6486

An interested reader drew to our attention that the western blots featured in Figs. 1B and 6C contained strikingly similar protein bands, and repeating patterns of bands, comparing across the lanes of the gels. Furthermore, an image representing the Myc-YAP colony-formation assay experiment in Fig. 2C was strikingly similar to the data shown for the Control colony-formation assay experiment in Fig. 5B.

The Editorial office subsequently investigated this matter further, and noted that the western blots shown in Fig. 6A and B likewise contained strikingly similar bands that were purportedly showing the results from different experiments. After having considered the various issues that have been brought to light with this paper, together with an appeal from the authors that a Corrigendum be published, the Editor of *Oncology Reports* has ruled that the article should be retracted from the publication on account of a lack of overall confidence in the presented data. Note that the authors were not in agreement that the errors reported and identified were sufficient to merit the retraction of the article. The Editor apologizes to the readership for any inconvenience caused.

